# Supplementary material for: Capturing COVID-19 spread and interplay with multi-hop contact tracing intervention
Source: PLoS One. 2023 Jul 13;18(7):e0288394. doi: 10.1371/journal.pone.0288394 (PMC10343086; doi:10.1371/journal.pone.0288394)
Supplement: S1 Text — (PDF) [file pone.0288394.s001.pdf]

Supplementary Information

S1 Text. Probability of detection for k-hop contact tracing

We compute the probabilities that a node i is detected under k-hop contact tracing for arbitrary k hops. We define terminologies for k-hop neighborhood (i.e., neighborhood within radius k) from a source node i in the original graph. The k-hop neighborhoods of node i is defined as the set of nodes that are reachable from the source node i in k hops or fewer. Denote $G_i^{(k)}(V_i^{(k)}, E_i^{(k)})$ as the sub-graph consisting of a set $V_i^{(k)}$ of nodes (the k-hop neighborhood of node i as well as node i) and a set $E_i^{(k)}$ of edges (all the edges between them). As in 2-hop contact tracing discussed in main manuscript, in order to compute the approximate probability, we need to convert the undirected cyclic graph, $G_i^{(k)}(V_i^{(k)}, E_i^{(k)})$, into an undirected acyclic graph, $\overline{G}_i^{(k)}(\overline{V}_i^{(k)}, \overline{E}_i^{(k)})$.

Let $\Delta_i^{(k)}$ denote the set of nodes at exactly k-hop from a source node i (i.e., $\Delta_i^{(k)} := V_i^{(k)} \backslash V_i^{(k-1)}$ where $V_i^{(0)} = i$). For k-hop contact tracing, we first traverse from the source node i to its direct neighbors, $\Delta_i^{(1)}$. We then traverse from each node of the set $\Delta_i^{(1)}$ to reachable nodes in the set of $\Delta_i^{(1)} \cup \Delta_i^{(2)}$. We repeat this process, and as a last step, we traverse from each node of the set $\Delta_i^{(k-1)}$ to reachable nodes in the set of $\Delta_i^{(k-1)} \cup \Delta_i^{(k)}$. During this process, we can visit the previously visited nodes again.

Recall that $\Pi_i^{X \to D}(t)$ is the probability that a node i in the state X is detected at time t and that the probability of detection for 2-hop is

$$\Pi_i^{X \to D}(t) \approx \begin{cases} 1 - \prod_{j \in \overline{V}_i^{(1)} \backslash \overline{V}_i^{(0)}} \left[1 - \overline{A}_{i:ij}^{(1)} f \rho_j^D(t) - \overline{A}_{i:ij}^{(1)} f \left\{1 - \rho_j^D(t)\right\} \left\{1 - \prod_{k \in \overline{V}_i^{(2)} \backslash \overline{V}_i^{(1)}} \left(1 - \overline{A}_{i:jk}^{(2)} f \rho_k^D(t)\right)\right\}\right], & X \in \{I_p, I_a\} \\ 1 - (1-\omega) \prod_{j \in \overline{V}_i^{(1)} \backslash \overline{V}_i^{(0)}} \left[1 - \overline{A}_{i:ij}^{(1)} f \rho_j^D(t) - \overline{A}_{i:ij}^{(1)} f \left\{1 - \rho_j^D(t)\right\} \left\{1 - \prod_{k \in \overline{V}_i^{(2)} \backslash \overline{V}_i^{(1)}} \left(1 - \overline{A}_{i:jk}^{(2)} f \rho_k^D(t)\right)\right\}\right], & X = I_s. \end{cases} \quad (1)$$

Let $\overline{A}_i^{(k)} = (\overline{A}_{i:jk}^{(k)})$ denote the adjacency matrix of $\overline{G}_i^{(k)}$. The probability, $\Pi_i^{X \to D}(t)$, for 3-hop contact tracing can be calculated using the acyclic graph assumption:

$$\Pi_i^{X \to D}(t) \approx \begin{cases} 1 - \prod_{j \in \overline{V}_i^{(1)} \backslash \overline{V}_i^{(0)}} \left[1 - \overline{A}_{i:ij}^{(1)} f \rho_j^D(t) - \overline{A}_{i:ij}^{(1)} f \left\{1 - \rho_j^D(t)\right\} \underbrace{\left[1 - \prod_{l \in \overline{V}_i^{(2)} \backslash \overline{V}_i^{(1)}} \left[1 - \overline{A}_{i:jl}^{(2)} f \rho_l^D(t) - \overline{A}_{i:jl}^{(2)} f \left\{1 - \rho_l^D(t)\right\} \left\{1 - \prod_{k \in \overline{V}_i^{(3)} \backslash \overline{V}_i^{(2)}} \left(1 - \overline{A}_{i:lk}^{(3)} f \rho_k^D(t)\right)\right\}\right]\right]}_{\text{Adaptation from equation } \Pi_i^{X \to D}(t) \text{ for 2-hop}}\right], & X \in \{I_p, I_a\} \\ 1 - (1-\omega) \prod_{j \in \overline{V}_i^{(1)} \backslash \overline{V}_i^{(0)}} \left[1 - \overline{A}_{i:ij}^{(1)} f \rho_j^D(t) - \overline{A}_{i:ij}^{(1)} f \left\{1 - \rho_j^D(t)\right\} \underbrace{\left[1 - \prod_{l \in \overline{V}_i^{(2)} \backslash \overline{V}_i^{(1)}} \left[1 - \overline{A}_{i:jl}^{(2)} f \rho_l^D(t) - \overline{A}_{i:jl}^{(2)} f \left\{1 - \rho_l^D(t)\right\} \left\{1 - \prod_{k \in \overline{V}_i^{(3)} \backslash \overline{V}_i^{(2)}} \left(1 - \overline{A}_{i:lk}^{(3)} f \rho_k^D(t)\right)\right\}\right]\right]}_{\text{Adaptation from equation } \Pi_i^{X \to D}(t) \text{ for 2-hop}}\right], & X = I_s. \end{cases} \quad (2)$$

As in the case of 2-hop, the terms in the large bracket $[\cdots]$ represent the probability that node i is not detected by any node in the particular branch including node j. Thus, $1 - \prod_{j \in \overline{V}_i^{(1)} \backslash \overline{V}_i^{(0)}} [\cdots]$ represent the probability that node i is detected by any of the nodes within radius 3 through 3-hop contact tracing. The second term in the bracket represents the probability that node i is detected by node j (1-hop neighbor). The third term represents the probability that node i is not detected by node j (1-hop neighbor) but is detected by any of the node's 2-hop or 3-hop neighbors in the particular branch including node j. The terms in the medium bracket $[\cdots]$ (inside the large bracket $[\cdots]$) represent the probability that node j is detected by any of node i's 2-hop or 3-hop neighbors. Thus, the terms in the medium bracket $[\cdots]$ (inside the large bracket $[\cdots]$) has the same form as the probability of detection for 2-hop (Equation 1), but only the superscript values increase by 1. Likewise, for 4-hop, this terms is replaced by the form of the probability of detection for 3-hop (Equation 2), but only the superscript values increase by 1. We can generalize to k-hop using the same process.
