# Supplementary material for: Capturing COVID-19 spread and interplay with multi-hop contact tracing intervention
Source: PLoS One. 2023 Jul 13;18(7):e0288394. doi: 10.1371/journal.pone.0288394 (PMC10343086; doi:10.1371/journal.pone.0288394)
Supplement: Attachment — Submitted filename: Response to Reviewers.pdf [file pone.0288394.s002.pdf]

Responses to the reviewer comments on "Capturing Epidemic Spread and Interplay with Multi-hop Contact Tracing Intervention"

Jungyeol Kim, Shirin Saeedi Bidokhti, Saswati Sarkar

We thank the reviewers for providing insightful comments and constructive suggestions which were of great help in improving the manuscript. We have revised our paper to address the reviewers' comments and suggestions. Our detailed response to comments follows (the comments from the reviewer are in *italics*). We point to the changes we have made in the manuscript in accordance with each comment. We reproduce the modifications from the manuscript as well for ease of reference. We have marked in blue the parts of the manuscript that we have added, modified, or moved to a different location, in response to the comments in the reviews.

I. Response to Reviewer #1

Comment 1. *Watts-Strogatz networks are the only synthetic networks chosen. Though Watts-Strogatz networks have similar average path lengths and clustering coefficients, their degree distributions are unrealistic and fail to produce the classic hub and spoke structure that real life networks have. Consequently, the reviewer urges the authors to additionally add Barabasi-Albert networks, which possess such hub and spoke property, to their experimental test bench. This would strengthen the authors' argument.*

Answer 1. We have now performed several additional computations for scale-free network (generated by Barabási-Albert model) to capture the existence of hubs with an excessive number of connections. We have presented the relevant findings in the revised manuscript (Figures 3 and 4 and discussions Section III-A) which we reproduce in Figures 4 and 5 in this response for convenience of the reviewer. We also reproduce the relevant discussions in the response to the reviewer's third comment.

Comment 2. *The entire work assumes that testing is 'free' and does not cost much. Though In Section III B 2., the authors argue that mass quarantining is much more expensive than mass testing, which the reviewer agrees with, the general assumption throughout the work is that testing capacity is virtually unlimited and easily scaled up which is unrealistic. Once the number of infections reaches critical mass, one is essentially testing the entire network which is not feasible. In the reviewer's opinion, this is the single biggest weakness that holds back this paper. The main idea behind this work is that multi-hop contact tracing is better than single hop contact tracing. To show that, a valid comparison between the two approaches needs to be done. This requires the number of tests in both cases to be roughly equal or otherwise it is trivially obvious that the one that uses more tests will do better. To address this issue, the reviewer suggests two approaches. Any, or preferably both of these approaches, if included would significantly strengthen this paper.*

1) *Compare the multi-hop approach to a baseline that uses a similar number of tests for an accurate comparison. For eg., you could compare the 2-hop approach with a 1-hop approach and random tests to make up the difference for the 1-hope approach. A few more less trivial procedures where the multi-hop approach performs better would strengthen this paper.*
2) *Set a budget for the number of tests that is quite small compared to the size of the network and compare the performance of 1-hop and multi-hop approaches given that budget. This is very similar to the minimal sensor set on networks problem that has been well explored for both epidemic forecasting and other applications.*

Answer 2: In view of the reviewer's comment, we have examined if the benefits of 2-hop contact tracing are only or primarily because it tests a larger number of individuals or because it intelligently selects who to test. As part of this examination, we have generalized our numerical computation framework two-fold and have described the generalizations in the newly created Section IV. The first generalization allows us to numerically evaluate a combination of k-hop contact tracing and additional random testing (Section IV-A in the revised version). This generalization allows us to approximately equalize the overall number of tests of 1) 2-hop contact tracing and 2) the combination of 1-hop contact tracing and additional random testing by choosing the number of additional random tests in the latter. This generalization therefore helps adopt the first suggestion of the reviewer. Once the overall number of tests have been equalized we compute and compare the outbreak size under each; the comparison reveals that 2-hop contact tracing still substantially reduces the outbreak size as compared to the combination, which in turn suggests that 2-hop contact tracing is able to significantly reduce the outbreak size because it judiciously deploys its additional tests as compared to 1-hop contact tracing. We subsequently obtain a realistic generalization wherein once an individual tests negative he does not test further for a certain duration unless he develops symptoms in the interim period;

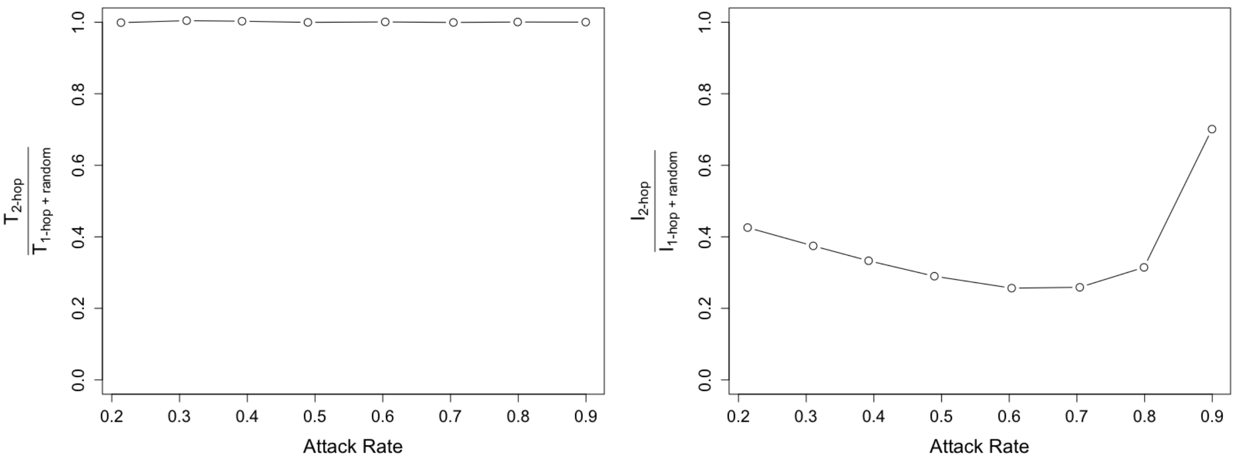

(a) (b)

Fig. 1. (a) The ratio of number of tests via 2-hop contact tracing to that under the combination of 1-hop contact tracing and random testing, $\frac{T_{2\text{-}hop}}{T_{1\text{-}hop+\text{random}}}$. (b) The ratio of cases infected via 2-hop contact tracing to the cases infected via '1-hop + random testing', $\frac{I_{2\text{-}hop}}{I_{1\text{-}hop+\text{random}}}$. We use the data-driven network for this figure.

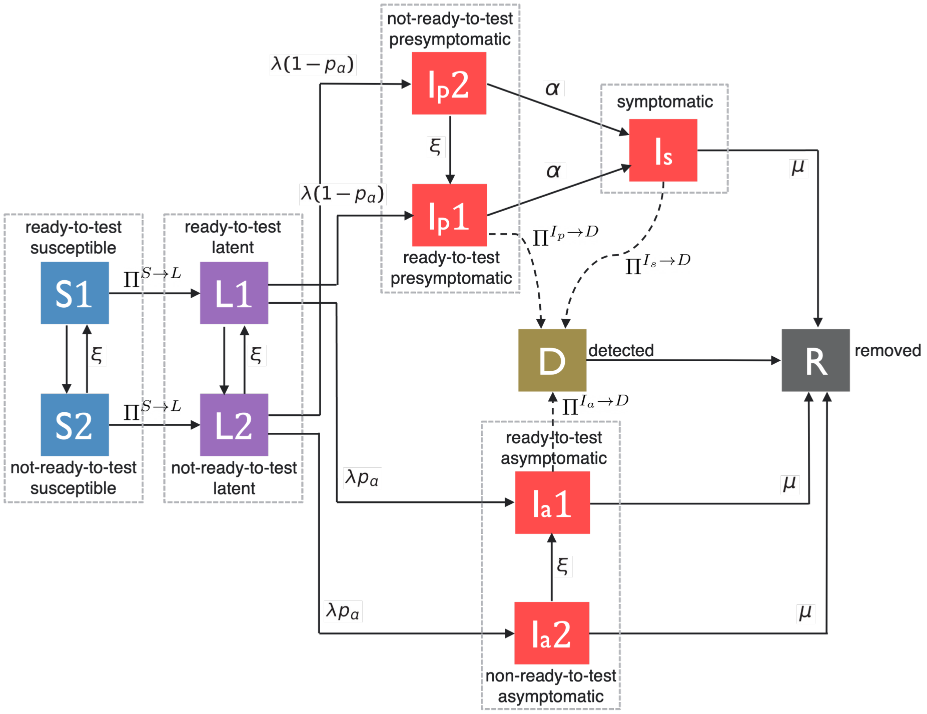

Fig. 2. A pictorial illustration of state transitions when there is test fatigue.

the size of the duration can be appropriately selected to satisfy budgets on average daily overall test count (Section IV-B in the revised paper). Broadly, this generalization incorporates the second suggestion of the reviewer. Our numerical computation reveals that 2-hop contact tracing continues to considerably reduce the outbreak size as compared to 1-hop contact tracing even when it satisfies stringent budgets on average daily overall test count and even as its number of tests becomes close to that for 1-hop contact tracing. This again shows that 2-hop contact tracing attains better cost-benefit tradeoff than 1-hop contact tracing because it judiciously selects which nodes to test. Both these generalizations demonstrate that our numerical computation framework is flexible enough to accommodate several features of reality. We describe the salient features of each generalization below.

Answer 2-1. We briefly describe how we adopt the first suggestion of the reviewer

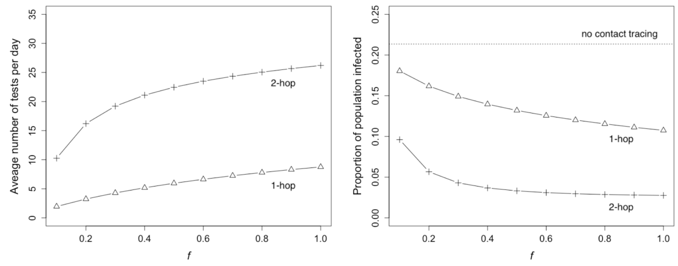

(a) $\xi = 1/14$

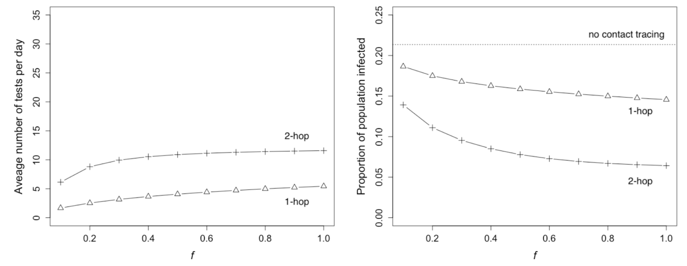

(b) $\xi = 1/60$

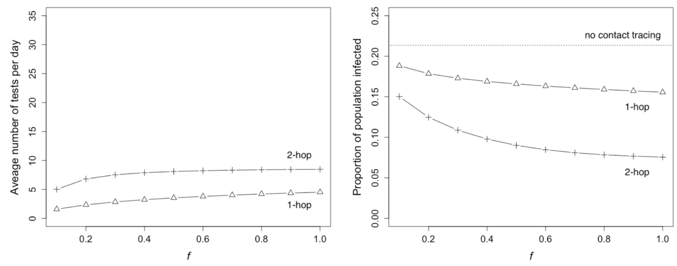

(c) $\xi = 1/90$

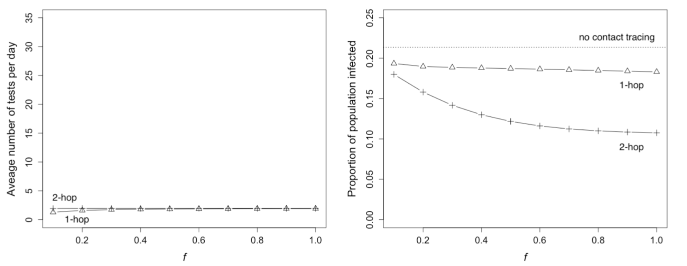

(d) $\xi = 0$

Fig. 3. The average number of tests per day and proportion of population infected via 1-hop and 2-hop as a function of activation probability f. Figures (a), (b), (c), and (d) show the results for $\xi = 1/14$, $\xi = 1/60$, $\xi = 1/90$ and $\xi = 0$, respectively. The horizontal dotted lines in the figures on the right represent the number of population infected when no contact tracing is performed. We use the data-driven network for this figure.

'2-hop' vs. '1-hop + random testing': In the revised version we show how the numerical computation framework can be generalized to incorporate a combination of contact tracing and random testing. We now assume that a node can be tested with probability δ for each time step even if he is not in k-hop neighborhood of a detected node or even if he does not show symptoms. Specifically, earlier pre-symptomatic and asypmtomatic nodes could be detected only through k-hop contact testing; now such nodes can be detected either through random testing or through k-hop contact tracing. Earlier symptomatic nodes could be detected through k-hop contact tracing, or testing with probability ω because of showing symptoms; now they may also be detected through random testing. Refer to Section IV-A of the revised manuscript for details on model equations in this case.

Considering $k = 1$ in the above framework, we control δ so that the total number of tests for '2-hop' and '1-hop + random testing' are almost the same (i.e., $\frac{T_{2\text{-}hop}}{T_{1\text{-}hop+\text{random}}} \simeq 1$). We then compare the total number of infections for '2-hop' and '1-hop + random testing'. Figure 1 shows that, despite both 2-hop' and '1-hop + random testing' incurring the same number of tests, 2-hop contact tracing provides significant advantage over '1-hop + random testing' strategy in terms of outbreak size for all attack rates. Recall that the attack rate is defined as the proportion of individuals in a population who has been infected in the time horizon under consideration, when no contact tracing is performed, which characterizes the intrinsic speed of virus spread in the absence of contact tracing.

Beyond adopting the first suggestion of the reviewer, we study a realistic generalization of our strategies which reduces the average daily number of tests of both 1-hop and 2-hop strategies and thereby satisfies budgetary constraints on this average through appropriate selection of parameters.

<u>Answer 2-2</u>.

Limiting the number of tests through a realistic generalization of our model: In the original manuscript, for the sake of model simplicity, we had assumed that individuals can be repeatedly tested if traced from contacts who test positive regardless of the outcome of their previous tests. But in reality, individuals develop a testing fatigue if they are frequently subjected to repeated tests, and the tests are negative. Due to this fatigue, individuals may therefore refuse to be tested shortly after a negative test, unless they develop symptoms, even if traced from contacts who test positive. Public health bodies may also avoid recommending tests for an individual shortly after he tests negative unless he develops symptoms, to avoid testing fatigue, elicit his cooperation in future, and satisfy constraints on the overall number of tests. We therefore consider that an individual is not tested for a random duration after he is tested negative unless he develops symptoms, even if he is traced from a 1-hop or 2-hop contact; if he tests positive he is quarantined and not tested again as we don't consider reinfection. The random duration is geometrically distributed with mean $1/\xi$. In Section IV-B of the revised manuscript, we describe how our model can be generalized to incorporate this variation for k-hop contact tracing strategies.

In Section IV-B of the revised manuscript, we also show that ξ can be tuned to have both 1-hop and 2-hop satisfy budgetary

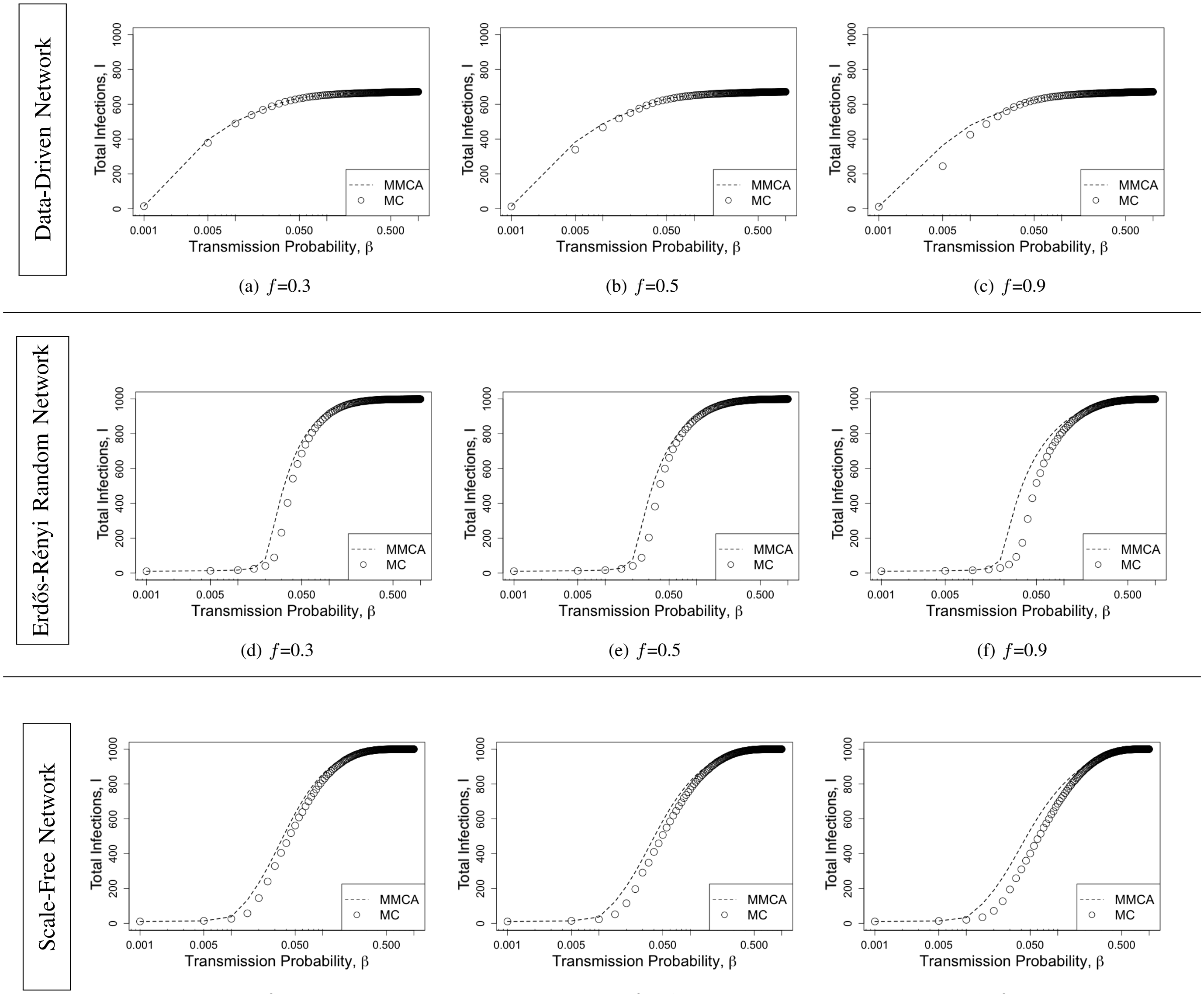

(a) f=0.3 (b) f=0.5 (c) f=0.9

(d) f=0.3 (e) f=0.5 (f) f=0.9

(g) f=0.3 (h) f=0.5 (i) f=0.9

Fig. 4. The total infection count I as a function of $\beta \in [0.001, 1]$ for 1-hop contact tracing for various contact networks. The first, second, and third rows respectively represent Data-Driven network, Erdős-Rényi Random Network, and Scale-Free Network. For each network topology, we consider various values of activation probability $f = 0.3, 0.5, 0.9$. The lines represent the MMCA formulation and the points represent MC simulation. The MMCA formulation closely approximates the MC simulation results under 1-hop contact tracing.

constraints on daily average number of tests, reduce the difference between the daily average number of tests of 2-hop and 1-hop, and still reduce the outbreak size through 2-hop compared to 1-hop. We reproduce below some of the salient points and figures in this response for ease of reading.

We consider several values of ξ, for an example attack rate, 0.21. We first assume that an individual who tests negative can be tested again after 14 days on average ($\xi = 1/14$), which is reasonable. This corresponds to a situation where average number of tests per day is less than 27 for 2-hop and 1-hop. Considering that the population size is 672, the average daily testing load is less than less than 4% of the total population. However, as shown on the right in Figure 3a, 2-hop can reduce the outbreak size by up to 73% compared to 1-hop and 87% compared to 0-hop. When $\xi = 1/60$, 2-hop can reduce the outbreak size by up to 55% compared to 1-hop and 69% compared to 0-hop (the right in Figure 3b). When $\xi = 1/90$, 2-hop can reduce the outbreak size by up to 51% compared to 1-hop and 64% compared to 0-hop (the right in Figure 3c). As an example of extreme case, to further constrain the testing budget, we assume that $\xi = 0$, i.e., individuals who have been tested once will not be tested again. This corresponds to a situation where the average number of tests per day is less than 2, and the difference in the number of tests between 1-hop and 2-hop becomes insignificant (the left in Figure 3d). Nevertheless, the right in Figure 3d shows that 2-hop can reduce the outbreak size by up to 41% compared to 1-hop and 49% compared to 0-hop.

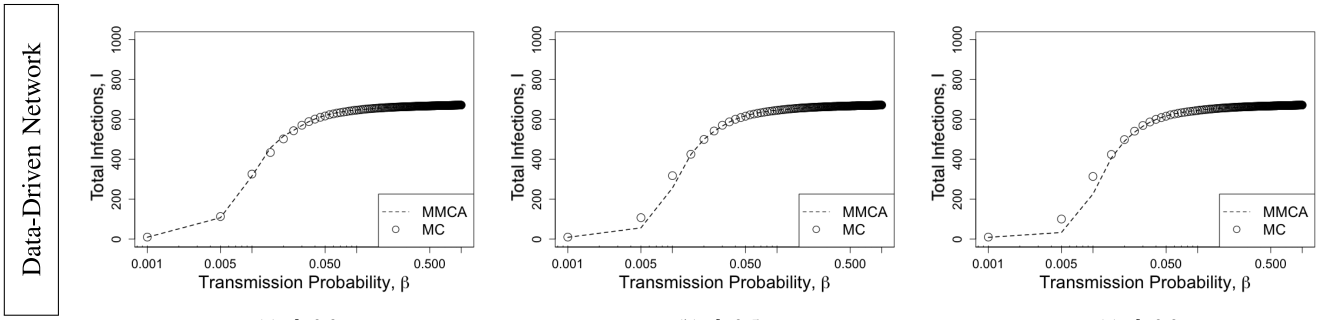

(a) f=0.3 (b) f=0.5 (c) f=0.9

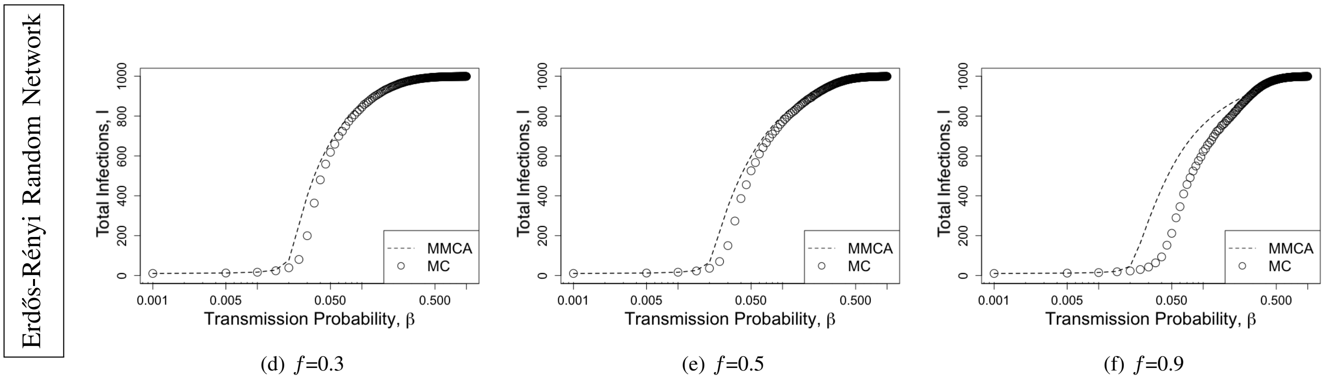

(d) f=0.3 (e) f=0.5 (f) f=0.9

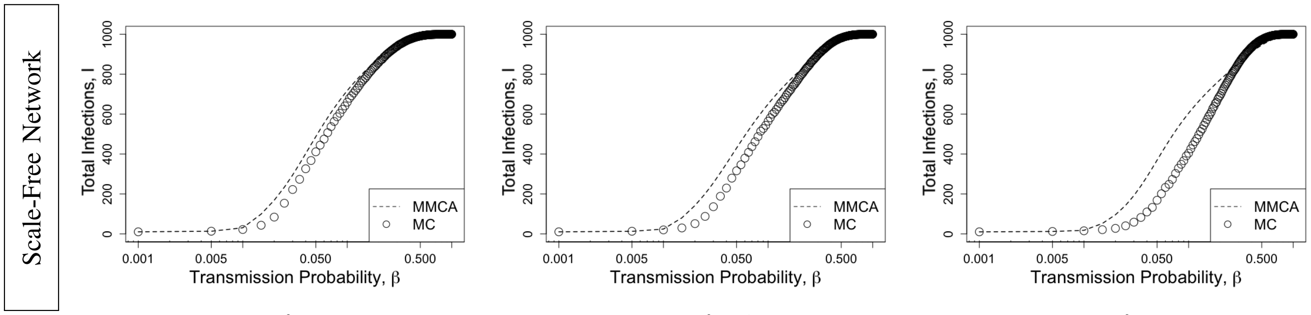

(g) f=0.3 (h) f=0.5 (i) f=0.9

Fig. 5. The total infection count I as a function of $\beta \in [0.001, 1]$ for 2-hop contact tracing for various contact networks. The first, second, and third rows respectively represent Data-Driven network, Erdős-Rényi Random Network, and Scale-Free Network. For each network topology, we consider various values of activation probability $f = 0.3, 0.5, 0.9$. The lines represent the MMCA formulation and the points represent MC simulation. The MMCA formulation closely approximates the MC simulation results under 2-hop contact tracing.

In conclusion, one can satisfy desired constraints on the average number of tests per day by appropriately choosing ξ. As ξ decreases average number of tests per day decreases for both 1-hop and 2-hop, and the average number of tests for both become closer and closer. Also, as ξ decreases, both 1-hop and 2-hop become closer to that for 0-hop. Nonetheless, for all values of ξ considered above, one can limit the average daily tests for both 1-hop and 2-hop under reasonable values and still attain a considerable reduction in outbreak size through 2-hop contact tracing over 1-hop contact tracing.

Comment 3. *It is difficult to interpret the figures. Though the results are clearly positive, it is not clear how to interpret the figures present. For eg., in Figures 2 and 3 it is not immediately clear which network the experiment was run on. Additionally, in Figure 7, it is not clear which figure is better at first glance. The reviewer suggests that the authors improve the figure descriptions and the figures themselves for easier reading.*

Answer 3. We regret the inconvenience. Following the reviewer's suggestion, we have now mentioned the network topology for each figure either on the side of the figure or in the captions. We have also reconstructed Figures 2 and 3 in the previously submitted manuscript, along with new plots for scale-free networks, to sharpen the presentation of the results. All the plots have been recreated into a total of two figures, broken down by types of contact tracing. Figures 4 and 5 here (Figures 3 and 4

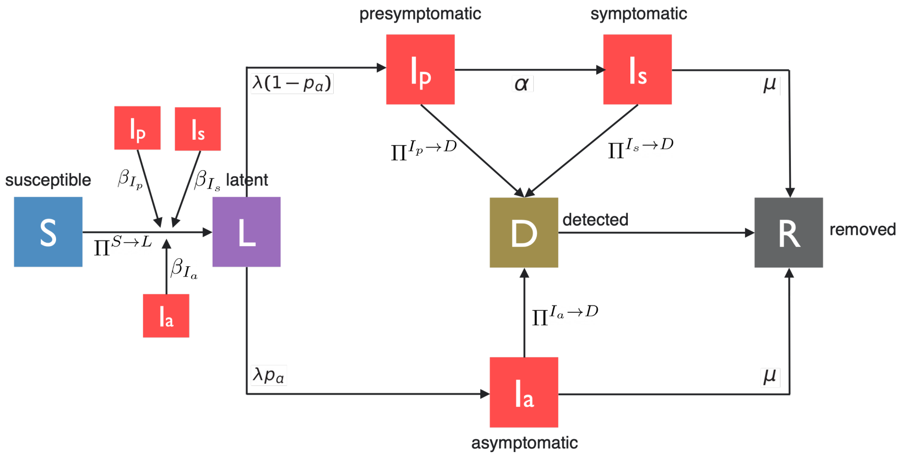

Fig. 6. A pictorial illustration of state transitions involved in the spread and evolution of COVID-19

in the revised manuscript) correspond to 1-hop and 2-hop contact tracing, respectively. Their descriptions have been reworded and the legends have been simplified for clearer interpretation. Refer to Section III-A (pp. 8-12) of the revised manuscript for corresponding text revised. We have improved the description of Figure 7 in the original manuscript, which is Figure 8 in the revised manuscript. Refer to the second paragraph of Section III-B-1 (p. 13) in the revised manuscript. The text has been reproduced below for easy reading.

> "We now try to understand why 2-hop contact tracing has a significantly lower infection count as compared to 1-hop contact tracing. For this we plot the total number of infections (I), the total number of detected infections (D), and undetected infections (U) over the course of 1 year (i.e., $\tau = 365$) for the two contact tracing strategies (Figure 8). We plot I, D, U as functions of activation probability f when attack rate is 0.8. The number of infections slowly decreases under 1-hop contact tracing as f increases, but there are still non-negligible number of undetected infections even in an ideal scenario of $f = 1$. The undetected infections continue to spread the infection leading to a large overall infection count. On the other hand, in case of 2-hop contact tracing, even a small increase in f can significantly reduce the number of undetected infections to almost zero. Thus infections are being detected and consequently isolated (quarantined). Thus not many nodes can spread the infection, which consequently significantly reduce the number of infections with even a small increase in f. It is clear that 2-hop contact tracing can effectively contain the epidemic even in challenging environments with a small f, primarily because it can detect and therefore isolate most of the infected individuals."

Comment 4. *The notation when reintroduced late, like in Section III B 2., results in significantly harder reading. The reviewer was repeatedly turning back to look up the notation while reading through. The reviewer urges the author to simplify the notation and add more explanations for the model formulation in Section II for easier reading.*

Answer 4. We have enhanced readability through the following changes: 1) We have now moved the definitions of notations in Section III of the original manuscript to Section II-B of the revised manuscript. Section II-B directly follows the expressions for probabilities of states and state transitions derived in Section II-A. Section II-B has been newly created in the revised manuscript. 2) We have also summarized the notations in notation Tables, and have separated them into one table for disease parameters (Table I in the revised manuscript) and another for evaluation metrics (Table II in the revised manuscript). 3) We have added several explanations throughout Section II. Refer to the text colored blue in the revised manuscript. 4) We have added a figure to pictorially illustrate the state transitions and the transition probabilities (Figure 6 reproduced in this response and Figure 1 in the revised manuscript).

II. Response to Reviewer #2

Comment 1. *Many of the references refer to the coronavirus pandemic, and I have the impression that this paper is within this context as well. It might be beneficial to explicitly state that in the introduction and/or abstract title.*

Answer 1. Following the reviewer's suggestion, we have changed the title to "Capturing COVID-19 Spread and Interplay with Multi-hop Contact Tracing Intervention". We have now mentioned COVID-19 in the first sentence of the abstract. We have also added the following sentence at the end of the Introduction:

> "Throughout the paper we consider the specific context of COVID-19, though our formulation can be adapted to other contagious diseases that spread through contact by choosing the appropriate compartmental model for the disease."

Comment 2. *In the model formulation, 3rd paragraph: I would add a little bit more explanation to clarify what omega is. Is it that a symptomatic infectious individual (I_s) gets tested with probability omega and when tested you assume that the test comes positive with probability 1? Also, you have not discussed about false positive and false negative tests. If you make the assumption that tests are 100% accurate, then you should state this.*

Answer 2. We have now explicitly stated several assumptions and have also clarified some concepts and notations.

- We have added the following paragraph in Section II (3rd paragraph of Section II):

  > "When an individual tests positive, he is said to be detected and enters the detected (D) state and remains there subsequently. A detected individual is quarantined until he is no longer contagious, ie, until he either recovers from the disease or is dead. Thus detected individuals can not infect others and are effectively removed from the system. Note that we do not consider re-infection in this paper. We consider that every test is accurate, ie, there are no false positives and no false negatives. Thus, whenever tested 1) presymptomatic, asymptomatic and symptomatic individuals test positive and are detected 2) susceptible and latent individuals test negative and are not detected (susceptible individuals test negative because they are not infected and since there is no false negative, latent individuals are already infected but the virus has not grown in them to the point that it can be detected through tests). Presymptomatic and asymptomatic individuals can be tested only as a result of contact tracing as they do not display symptoms. Symptomatic individuals can be tested either 1) after being traced from a contact or 2) because they develop symptoms. We consider that a symptomatic individual is tested because he develops symptoms with probability ω, ω can be less than 1 as not everyone reports his symptoms."

- We have now organized the notations into two new tables, one for disease parameters (Table I in the revised manuscript) and another for evaluation metrics (Table II in the revised manuscript). We have now included the definition of ω in Table I.

Comment 3. *A diagram might be beneficial to the reader, showing the different states and arrows connecting them with the relevant probabilities. For example $S \rightarrow L$ with $+\ I_s\beta_{I_s}$ on top of the arrow, and two more arrows for β_{I_p} and β_{I_a}. Then under L you can have $1/\lambda$ to show the duration.*

Answer 3. Following the reviewer's suggestion, we have included a figure showing the state transitions and relevant probabilities. Please refer to Figure 6 here (Figure 1 in the revised manuscript, p. 3).

Comment 4. *Typo in the 6th equation of the set of equations showing the evolution of probabilities (page 3): 2nd term of right hand side subscript of rho should be I_a.*

Answer 4. We have now corrected the typo (p. 4). We apologize for our error.

Comment 5. *For added clarity of eq.1 I would add some more explanation, eg prob of node i staying in S state is $1 - \Pi_i^{S\rightarrow L}(t) = \Pi_{j=1}^{N}$ [prob of node i not getting infected via contact with node j]. This will make the paper more accessible to readers not specialising in epidemic modelling, and set the basis for the rest of the paper. In eq.2 you need to state clearly that this is the one with no contact tracing. Again I would add a small paragraph to explain eq. 3.*

Answer 5. We have added the explanations the reviewer suggests. Please refer to the text marked in blue in p. 4.

Comment 6. *Page 5 paragraph starting with 'To verify': Referal to eq. (13) in line 2 and last line of that paragraph.*

Answer 6. We have corrected the equation number (13) to (4) in the manuscript. Refer to the text marked in blue in penultimate paragraph of Section II-A, p. 5. We are sorry for the confusion caused by this unfortunate error.

Comment 7. *Page 5 paragraph starting with 'Next, we compute' you have the sentence: 'Individuals in symptomatic (Is) state can be tested and detected either after showing symptoms or via contact tracing; thus, the probability that an individual in symptomatic (Is) state is tested at time t is equivalent to the probability that the person is detected at time t.' Here you are*

assuming 100% accuracy of test, right? It would be beneficial to state this.

Answer 7. We have now stated our assumption on accuracy of tests in the revised manuscript. Please refer to our response to your Comment 2.

Comment 8. *Eq. 6 please add an explanation of the last term - why does omega appear there? This goes along with my second comment on clarifying what omega is.*

Answer 8. We appreciate the reviewer for pointing this out. We had inadvertently included ω in the last term. We have now rectified our error. The corrected equation is:

$$T(t) = \sum_{i=1}^{N} \left[\Pi_i^{S\to T}(t)\rho_i^S(t) + \Pi_i^{L\to T}(t)\rho_i^L(t) + \Pi_i^{I_a\to T}(t)\rho_i^{I_a}(t) + \Pi_i^{I_p\to T}(t)\rho_i^{I_p}(t) + \Pi_i^{I_s\to T}(t)\rho_i^{I_s}(t) \right].$$

This constitutes the equation after equation (10) in Section II-B-1, p. 7 of the revised manuscript. We had used the above equation only to compute the data plotted in Figure 12. We have now replotted the Figure with the corrected equation. Despite this change, the new figure (Figure 12, p. 16 of the revised manuscript) is not qualitatively different from the old figure (Figure 11 in the original submission).

Comment 9. *Page 6: It might be beneficial to the reader to explain some terminology: Watts-Strogatz, average path lengths, clustering coefficients.*

Answer 9. We reproduce the reworded content for the reviewer's easy reference.

Reworded description on terminology about networks (Section III-A, 2nd paragraph, pp. 8-10):

> "We now describe the synthetic networks we consider in this section. Watts-Strogatz network is a synthetic network generated as follows. We start with a regular ring lattice and each endpoint of each edge is rewired with a certain probability to another node chosen uniformly randomly over the rest of the ring. We choose the Watts-Strogatz as representative of synthetic networks because two important attributes that determine the rate of spread of infectious diseases in contact networks, namely average path lengths, and clustering coefficients, can be controlled in this class through the choice of a parameter, namely the rewiring probability. Accordingly, this class of networks has been utilized extensively for simulation studies in spread and control of infectious diseases over contact networks [1]. Note that the average path length is defined as follows for a given network. The distance between a pair of nodes is the length of the shortest path between them, i.e., the number of links in the path between them that has the minimum number of links among all paths between them. The average path length for a given network is the average of this distance over all pairs of nodes in it. Clustering coefficient is the average of C_i over all nodes i where C_i is defined as the ratio between the actual number of links between the neighbors of i and the maximum possible number of links between the neighbors of i [2]. As the rewiring probability increases from 0 to 1, 1) average path lengths range from linear to logarithmic functions of the number of nodes 2) clustering coefficients range from high to vanishingly small [3], [2]. The special case of the Watts-Strogatz model in which the rewiring probability is 1 corresponds to a variant of the Erdős Rényi random networks which has also been extensively utilized in the study of spread and control of infectious diseases [4], [5]; we consider this variant as well. Furthermore, we consider scale-free network (generated by Barabási-Albert model) to capture the existence of hubs with an excessive number of connections. Unlike scale-free network, the probability of the existence of the hubs in Watts–Strogatz network is low."

Comment 10. *Page 7: Transmission probability beta now appears with no subscript. I guess you are assuming that* $\beta_{I_s} = \beta_{I_a} = \beta_{I_p}$*? If so, please state it. Is this realistic?*

Answer 10. In the Model Formulation section (Section II), we defined the three probabilities as different parameters for model flexibility. However, when evaluating the effectiveness of contact tracing in Section III, we assumed $\beta_{I_s} = \beta_{I_a} = \beta_{I_p} = \beta$ by following the parameter setup in study [6]. We have now explicitly mentioned as the first paragraph of Section III (p. 8). We reproduce the sentence below for ease of reading:

> "Throughout this section, we assume $\beta_{I_s} = \beta_{I_a} = \beta_{I_p}$ and denote all of these as β."

We have acknowledged the limitation of this assumption in the Conclusion and Discussion Section (4th paragraph of Section V, p. 22). We have added the following paragraph there:

"When evaluating the effectiveness of contact tracing in Section III, we had assumed that $\beta_{I_s} = \beta_{I_a} = \beta_{I_p} = \beta$ by following the parameter setup in study [6], although we had allowed these parameters to be different while formulating our model in Section II. In reality, different types of contacts may pass on infection with different probabilities. This transmission probability may also depend on a range of factors, such as whether the individuals observe social distancing and wear protective equipment, and varies from one venue to another. Explicitly investigating the impact of non-uniform transmission probabilities constitute directions for future research."

Comment 11. *Page 13 when discussing costs, what about the hospitalisation costs? Also, public health systems have limitations in hospitalisation capabilities, so relevant questions might be worth asking. I am not suggesting you add anything more to the current paper, but it might be worth discussing this in the discussion section.*

Answer 11. We have added the following paragraph in the Conclusion and Discussion Section (5th paragraph of Section V, p. 22):

"We have considered two public health cost metrics, quarantine cost and testing cost. There are several other cost metrics that are of interest from a public health perspective such as overall hospitalization and death counts. Hospitalizations are of interest particularly because public healthcare systems have limited hospitalization capacity. Hospitalization and death counts can be investigated by adding new states to the model e.g., a hospitalization state can be added between symptomatic and removed states, the hospitalization state can lead into dead and recovered states, etc. Comparing different contact tracing strategies from the point of view of those costs constitutes a direction of future research."

Comment 12. *When discussing the different strategies of no contact tracing, 1-hop or 2-hop contact tracing, it might be beneficial to consider the total number of contacts required to be traced per day. You do include the fraction f as an indication of a limitation to this, but in practice, wouldn't the contact tracing team have the limitation in the number of contacts traced per day, and not the fraction? Eg all the contacts can be traced at the beginning of the epidemic, but when it gets out of hand, the contacts of only 1000 detected people can be traced per day. I am not suggesting you add anything more to the current paper, but it might be worth discussing this in the discussion section.*

Answer 12. We have added the following paragraph in the Conclusion and Discussion Section (3rd paragraph of Section V, p. 22):

"We now describe some limitations of our work which in turn identify directions for future research. We have implicitly assumed that any number of contacts can be traced on any given day. But public health systems may have constraints on the number of contacts that can be traced per day owing to limitation on tracing personnel for example. There may be constraints on the average number of contacts that can be traced per day which can be satisfied through an appropriate choice of f. As f decreases, average number of contacts traced per day decreases. There may also be hard upper bound on the number of contacts that can be traced on each day. In this case, contact tracing strategies need to be adapted to satisfy such constraints. This constitutes a direction for future research. Such constraints however become less stringent if contacts are traced through digital tools, e.g, digital contact tracing apps. For example, digital tools were utilized in Vietnam to execute multi-hop contact tracing [7]. But then not all contacts can be digitally traced either as not everyone downloads such apps and the willingness is different in different countries. In Singapore, for example, more than 92% of the population aged six and above had downloaded the government's contact tracing app on their smartphones [8], but the percentage has been lower in other countries. The nature of the constraints depend on the degree of reliance of the public health system on manual tracing."

References

[1] Y. Pan and Z. Yan, "The impact of multiple information on coupled awareness-epidemic dynamics in multiplex networks," Physica A: Statistical Mechanics and its Applications, vol. 491, pp. 45–54, 2018.

[2] D. J. Watts and S. H. Strogatz, "Collective dynamics of 'small-world'networks," Nature, vol. 393, no. 6684, pp. 440–442, 1998.

[3] F. C. Santos, J. F. Rodrigues, and J. M. Pacheco, "Epidemic spreading and cooperation dynamics on homogeneous small-world networks," Physical Review E, vol. 72, no. 5, p. 056128, 2005.

[4] I. Z. Kiss, D. M. Green, and R. R. Kao, "Disease contact tracing in random and clustered networks," Proceedings of the Royal Society B: Biological Sciences, vol. 272, no. 1570, pp. 1407–1414, 2005.

[5] L. G. A. Zuzek, C. Buono, and L. A. Braunstein, "Epidemic spreading and immunization strategy in multiplex networks," in Journal of Physics: Conference Series, vol. 640, no. 1. IOP Publishing, 2015, p. 012007.

[6] A. Reyna-Lara, D. Soriano-Paños, S. Gómez, C. Granell, J. T. Matamalas, B. Steinegger, A. Arenas, and J. Gómez-Gardeñes, "Virus spread versus contact tracing: Two competing contagion processes," Physical Review Research, vol. 3, no. 1, p. 013163, 2021.

[7] P. Q. Thai, M. A. Rabaa, D. H. Luong, D. Q. Tan, T. D. Quang, H.-L. Quach, N.-A. Hoang Thi, P. C. Dinh, N. D. Nghia, T. A. Tu et al., "The first 100 days of severe acute respiratory syndrome coronavirus 2 (SARS-CoV-2) control in vietnam," Clinical infectious diseases, vol. 72, no. 9, pp. e334–e342, 2021.

[8] H. Baharudin, "More than 1,100 users have deregistered from TraceTogether: Vivian," The Straits Times, 2021, https://www.straitstimes.com/singapore/more-than-1100-users-have-deregistered-from-tracetogether-vivian (accessed 25 May 2021).
